# 

**Published:** 1917-03-17

**Authors:** 


### PROTECTION OF MILK SUPPLY.

A lecture by Dr. W. G. Savage was delivered at the Royal Institute of Public Health, Russell Square, W.C., on February 28. The lecturer outlined the extent to which improvements in the protection of the milk supply can be effected during the war, and the steps which should be introduced after peace has been re-established.

Rates of Subscription: Twelve months, 6/6; Six months, 4/-; Three months, 2/-; post free to any address in the United Kingdom. Abroad, Twelve months, 8/8; Six months, 5/-; Three months, 2/6, post free. The Hospital "Index" to Volume LX., April to September 1916, is now ready, price 2½d. per copy, post free. Address The Manager, "The Hospital," The Hospital Building, 28/29 Southampton Street, Strand, London, W.C.

